# Enhanced Liver Fibrosis Test, FIB‐4 and FibroScan: Real‐World Prognostic Accuracy for MASLD in a Biopsy‐Controlled Cohort

**DOI:** 10.1111/liv.70774

**Published:** 2026-07-02

**Authors:** Antonio Liguori, Francesca D'Ambrosio, Giulia Angelini, Consuelo Cefalo, Simone Galletti, Sara Cardinali, Maria Cristina Giustiniani, Giuseppe Marrone, Marco Biolato, Gianludovico Rapaccini, Maurizio Pompili, Antonio Grieco, Tommaso Mazza, Andrea Urbani, Maurizio Sanguinetti, Antonio Gasbarrini, FPG‐UCSC PROMETEO‐ELF substudy Research Group, Luca Miele

**Affiliations:** ^1^ Department of Medical and Surgery Sciences Fondazione Policlinico Universitario A. Gemelli IRCCS Rome Italy; ^2^ Department of Translational Medicine and Surgery Università Cattolica del Sacro Cuore Rome Italy; ^3^ Department of Laboratory and Hematology Sciences Fondazione Policlinico Universitario A. Gemelli IRCCS Rome Italy; ^4^ Women, Children and Public Health Sciences Fondazione Policlinico Universitario A. Gemelli IRCCS Rome Italy; ^5^ Computational Biology and Bioinformatics Unit Fondazione Policlinico Universitario A. Gemelli IRCCS Rome Italy

**Keywords:** ELF, Fibroscan, liver related events, MASLD, non‐invasive tests

## Abstract

**Background and Aims:**

Liver fibrosis is a major determinant of liver‐related events (LREs) in patients with metabolic dysfunction‐associated steatotic liver disease (MASLD). Effective stratification based on fibrosis severity is essential for accurate prognosis. The Enhanced Liver Fibrosis (ELF) test, a non‐invasive test measuring direct fibrosis markers, offers a potential alternative to traditional methods like FIB‐4, liver stiffness measurement (LSM) and liver biopsy. This study aims to evaluate the prognostic value of the ELF test in comparison with these established methods.

**Methods:**

We retrospectively analysed 289 MASLD patients (30.4% female, median age 49 years [IQR 39–58], 28% with type 2 diabetes) from the PROMETEO cohort (NCT04371042). We excluded patients with prior or current evidence of decompensated cirrhosis. At baseline, patients underwent liver biopsy, FIB‐4, ELF and LSM assessments. The primary outcome was development of Liver Related Events (LRE), a composite of liver‐related death, hepatocellular carcinoma, liver transplantation, ascites, encephalopathy or variceal bleeding.

**Results:**

Over a median follow‐up of 64 months [IQR 39–81], 41 patients (14.2%) experienced LREs. Event rates increased markedly across ELF categories (1% for ELF < 9.8, 27.8% for ELF ≥ 9.8 to < 11.3 and 72.7% for ELF ≥ 11.3). ELF demonstrated discriminative performance comparable to histological fibrosis stage and LSM, with the highest overall AUC among evaluated tests. In adjusted Cox models, higher ELF categories remained strongly associated with LRE risk, although confidence intervals were wide due to the limited number of events.

**Conclusions:**

In this biopsy‐proven MASLD cohort, ELF provided prognostic stratification for liver‐related events comparable to liver histology and LSM. Given the limited number of outcome events, these findings support the non‐inferiority of ELF rather than definitive superiority. Larger prospective studies are needed to confirm these results and refine risk prediction strategies.

## Lay Summary

- The Enhanced Liver Fibrosis (ELF) test, a simple blood test, predicts liver‐related complications in patients with MASLD demonstrating discriminative performance comparable to histological fibrosis stage and LSM in the real‐world setting.
- ELF effectively stratifies patients into low‐, intermediate‐ and high‐risk groups, helping identify those at greatest risk of severe outcomes.
- This non‐invasive test offers a reliable alternative to invasive procedures, improving patient care and monitoring in real‐world clinical settings.

**Abbreviations**
AUC: area under the receiving operating curve
ECM: extracellular matrix
ELF: enhanced liver fibrosis
HA: hyaluronic acid
HR: Hazard Ratio
LRE: liver‐related events
LRE: liver‐related events
LSM: liver stiffness measurement
MASLD: metabolic dysfunction‐associated steatotic liver disease
MMPs: matrix metalloproteinases
NFS: NAFLD Fibrosis Score
NITs: non‐invasive tests
PIIINP: amino‐terminal peptide of type 3 procollagen
SBP: spontaneous bacterial peritonitis
TIMP‐1: tissue inhibitor of metalloproteinases 1

## 1 Introduction

Metabolic dysfunction‐Associated Steatotic Liver Disease (MASLD) is increasingly common, afflicting at least 1 in 5 people living in Western nations [1]. A major focus of clinical care for patients with MASLD should be the determination of those at highest risk for the complications of advanced liver disease [2, 3]. Liver fibrosis is the most important risk factor for decompensated cirrhosis and liver cancer in patients with MASLD, and liver fibrosis severity is the strongest predictor of clinical outcomes [4, 5, 6]. Currently, liver biopsy represents the gold standard for staging fibrosis, although its limitations related to costs, invasiveness, repeatability, sampling errors and complications [7].

The rising prevalence of MASLD, along with the previously mentioned limitations of liver biopsy, has driven the development of various non‐invasive tests (NITs) for precise staging of liver disease and risk stratification. Several NITs have been proven to be accurate in detecting liver fibrosis, particularly in patients with MASLD. Among these, liver stiffness measurement (LSM), blood test biomarkers (enhanced liver fibrosis—ELF) and scores (Fibrosis‐4—FIB‐4 and NAFLD Fibrosis Score—NFS) are the most validated and widely used [7, 8, 9, 10, 11].

The ELF test is a multiparametric panel of direct fibrosis markers which measures three blood biomarkers reflecting ECM turnover: tissue inhibitor of metalloproteinases 1 (TIMP‐1), amino‐terminal peptide of type 3 procollagen (PIIINP) and hyaluronic acid (HA).

Considering the close correlation between ELF and liver fibrosis, this test was also proposed and assessed as a non‐invasive prognostic factor in patients with chronic liver disease. Indeed, the ELF test can predict liver‐related events (LRE) in the general population [12] and in patients with chronic liver disease related to HCV [13, 14], alcohol‐related liver disease [15] and cholestatic diseases [16, 17]. In MASLD patients, ELF test lower than 9.8 and higher than 11.2 identify patients at low and high risk to develop LRE, respectively [18, 19]. Despite the good prognostic ability demonstrated by ELF, it is unclear to what extent it can replace histological assessment of liver fibrosis in the prognostic assessment of patients with MASLD.

Younossi et al. [20] showed that in a population of 2154 patients enrolled in clinical trials and diagnosed with MASH and advanced fibrosis (Stage 3 or 4 at histological assessment), ELF can predict the risk of progression to liver cirrhosis (for patients with Fibrosis stage 3 at baseline) and the risk of LRE.

The aims of this study were as follows: (1) to investigate the ability of ELF to predict LRE in patients with MASLD and (2) to compare the prognostic ability of ELF, histologically assessed fibrosis and other NITs as part of real‐world clinical practice.

## 2 Material and Methods

### 2.1 Study Design and Patients

This is a single‐centre, biopsy‐controlled study based on the PROMETEO registry (NCT04371042). The study adheres to the TRIPOD checklist for transparent reporting of prediction models (Table S1) [21]. All patients signed a consent form.

We included patients who underwent liver biopsy from January 2011 to December 2022 at Steatotic Liver Disease (SLD) Outpatient Clinic at Fondazione Policlinico Universitario A. Gemelli in Rome, Italy. Liver biopsy was performed according to usual clinical practice in our centre and international EASL guidelines [22], on patients with ultrasound findings of liver steatosis and evidence of deranged liver function tests (ALT > 2× upper normal limits) and/or increased liver stiffness (liver stiffness measurement > 8 kPa—Fibroscan). The inclusion criteria were age 18–75 years and diagnosis of MASLD according to the latest Delphi consensus [23]. Exclusion criteria were prior or current evidence of decompensated cirrhosis (MELD > 15, Child‐Pugh B or C, ascites, overt hepatic encephalopathy and history of variceal bleeding), prior or current chronic alcohol overuse defined as > 24 g/day for women and > 36 g/day for men, concurrent liver disease other than MASLD (chronic viral hepatitis B or C, autoimmune or cholestatic liver diseases, Wilson's disease and hemachromatosis) and hepatic or extrahepatic cancer. Anthropometric data, clinical and laboratory parameters were collected at the time of liver biopsy.

### 2.2 Non‐Invasive Tests and Liver Biopsy

Fasting blood samples were collected from all patients at the time of biopsy and serum aliquots were obtained by centrifugation (3000 rpm × 10 min) and stored at −80°C. Assay of HA, PIIINP and TIMP‐1 were determined in accordance with the manufacturer's instructions, using the Atellica IM Analyser (Siemens Healthineers, US). The concentrations of each marker were reported in ng/mL, and the ELF test value was automatically calculated by the analyser with the algorithm: ELF score = 2.278 + 0.851 ln[HA] + 0.751 ln[PIIINP] + 0.394 ln[TIMP‐1], validated for Atellica IM/ADVIA Centaur (Siemens Healthineers, US) platforms and reagents. Serum dilutions were performed according to the manufacturer's recommendations when necessary. An operator (F.D.) unaware of clinical information performed the assays.

Liver stiffness was assessed by vibration‐controlled transient elastography by FibroScan (Echosens, Paris, France) at the time of liver biopsy [24]. We calculated FIB‐4 from routine blood tests performed at the time of liver biopsy [25].

Two experienced liver pathologists (M.C.G. and F.M.V.) assigned NAFLD Activity score and Kleiner fibrosis stage while unaware of all other study data. A minimum length of 10 mm and at least 6 portal tracts are required to consider a biopsy to be of adequate quality. Metabolic‐dysfunction associated steato‐hepatitis (MASH) was defined by (NAS ≥ 4 with ≥ 1 point in steatosis, inflammation and ballooning) [23, 26].

### 2.3 Liver Related Events (LRE)

Primary outcome, LRE, was defined as the occurrence during the follow up of either ascites, variceal bleeding, spontaneous bacterial peritonitis (SBP), MELD score ≥ 15, overt hepatic encephalopathy, hepatocellular carcinoma and liver related death. We included in the analysis two secondary outcomes: Acute Decompensation (AD) and Acute on Chronic Liver Failure (ACLF). AD was defined as by the acute development of > 1 of the following major complication(s): first grade 2 or 3 ascites, first episode of acute hepatic encephalopathy in patients with previously normal consciousness, SBP, acute gastrointestinal bleeding. We do not consider any infection as an AD according to a recent prospective study [27]. ACLF was defined according to EASL‐CLIF criteria and is characterized by the functional failure of one or more of the six major organ systems (i.e., liver, kidney, brain, coagulation, circulation and respiration), and systemic inflammation [28]. Patient's electronic medical record was reviewed to determine the date of the last recorded medical contact and to collect date and type of LRE, AD, ACLF, death or liver transplantation date. If the last recorded event was > 12 months before the date of ascertainment of follow‐up data, the patient was contacted by telephone (a maximum of three attempts).

### 2.4 Statistical Analysis

Baseline characteristics were compared between patients who developed the primary composite outcome and those who did not. Categorical variables were compared using *χ* ^2^ tests or Fisher's exact tests, as appropriate. Continuous variables were assessed for normality using the Kolmogorov–Smirnov test and compared using independent‐samples *t*‐tests or Mann–Whitney U tests, as appropriate. All tests were two‐tailed, and a *p* value < 0.05 was considered statistically significant.

The discriminative ability of NITs to predict the primary outcome was assessed using the area under the receiver operating characteristic curve (AUC). Time dependent AUCs were compared using Inverse Probability of Censoring Weighting (IPCW)‐weighted ROC curves and DeLong tests at pre‐defined landmarks (1, 3 and 5 years). *p* values were adjusted for multiple comparisons using the Benjamini–Hochberg procedure.

Patients were stratified into three risk categories according to pre‐defined cutoffs for ELF (< 9.8, ≥ 9.8 to < 11.3, ≥ 11.3), FIB‐4 (< 1.3 [2 in > 65 years], ≥ 1.3 [2 in > 65 years] to < 2.67, ≥ 2.67), liver stiffness measurement (LSM; < 10, ≥ 10 to < 15, ≥ 15 kPa) and histological fibrosis stage (F0‐2, F3, F4). A two‐step stratification strategy was also evaluated according to current evidence and clinical guidelines [22, 29], consisting of FIB‐4 assessment in all patients followed by LSM using the cut‐offs of 8 and 12 kPa. Low‐risk patients were defined as those with FIB‐4 < 1.3 (or 2.0 if age ≥ 65 years) or FIB‐4 ≥ 1.3 [or 2.0 if age ≥ 65 years] to < 2.67 combined with LSM < 8 kPa; high‐risk patients were defined as those with FIB‐4 > 2.67 or FIB‐4 ≥ 1.3 [or 2 if age ≥ 65 years] to < 2.67 combined with LSM > 12 kPa; the remaining patients were classified as intermediate risk. Kaplan–Meier curves were generated to evaluate time to the primary outcome stratified by ELF, LSM, FIB‐4 and histology, and the two‐step approach, and compared using log‐rank test. Cox proportional hazards regression was used to estimate hazard ratios (HRs) with corresponding 95% confidence intervals (CIs). Univariable Cox models were used to describe risk gradients across pre‐defined categories. Multivariable Cox regression analyses were performed primarily as sensitivity analyses and were restricted to a small set of pre‐specified clinically relevant covariates (age, sex, body mass index and type 2 diabetes) to limit overfitting given the number of events. No data‐driven variable selection was performed. For primary inference, ELF was also analysed as a continuous variable to maximize statistical power, and results were interpreted based on effect sizes and confidence intervals rather than sole reliance on *p* values.

Incidence rates were assessed and compared for each risk subgroup. Incidence rate ratios (IRRs) were used as descriptive measures to compare relative event rates across categories and were interpreted in conjunction with Kaplan–Meier analyses and discrimination metrics.

To account for potential confounding due to differences in baseline age and sex across ELF categories, inverse probability of treatment weighting (IPTW) was applied. A multinomial logistic regression model was used to estimate the probability of belonging to each ELF stratum conditional on covariates. Stabilized weights were then derived and applied to create a pseudo‐population in which the distribution of age and sex was balanced across ELF groups. Average treatment effects (ATE) and potential outcome means (POmeans) were estimated using the teffects ipw procedure in Stata (version 17, StataCorp LLC, Texas, USA), with robust variance estimators to account for weighting.

All statistical analyses were performed using R (version 4.0.2, R Foundation for Statistical Computing, Vienna, Austria) and STATA (version 17, StataCorp LLC, Texas, USA). A *p*‐value of < 0.05 was considered statistically significant.

## 3 Results

### 3.1 Demographics

We included 289 subjects with histologically confirmed MASLD across different stages of disease. Median age at inclusion was 49 years (IQR 39–58), 201 patients (69.6%) were male and 81 (28%) had type 2 diabetes at baseline (Table 1). One hundred forty‐seven patients (50.8%) fulfilled histological criteria for MASH, whereas 142 (49.2%) had MASLD with fibrosis but without meeting full MASH criteria. This latter group may include patients with ‘burnt‐out’ MASH who developed fibrosis despite resolution of necroinflammatory activity, or individuals who developed fibrosis in the presence of only low‐grade inflammation or hepatocyte ballooning.

**TABLE 1:** Baseline patients' characteristics, stratified for composite outcome.

	Total (*n* = 289)	Composite outcome during follow‐up		
		Yes *n* = 41	No *n* = 248	*p* *
Follow‐up time (months)	64.0 (39.0–81.0)	22.0 (11.0–42.0)	65.5 (48.0–83.0)	0.06
Gender (male)	201 (69.6)	25 (61.0)	176 (71.0)	0.27
Age (years)	48.8 (39.0–58.0)	63.6 (59.2–69.0)	47.0 (37.0–54.0)	< 0.01
Diabetes	81 (28.0)	25 (61.0)	56 (22.6)	< 0.01
Hypertension	110 (38.1)	21 (51.2)	89 (35.9)	0.09
BMI (Kg/m^2^)	28.7 (25.5–31.8)	27.3 (24.2–30.7)	28.7 (26.1–32.4)	0.05
ALT (IU/L)	52.0 (34.0–78.0)	44.0 (19.0–56.0)	53.5 (35.0–81.2)	< 0.01
AST (IU/L)	39.0 (27.0–54.0)	42.0 (27.0–59.0)	38.5 (27.0–54.0)	0.42
GGT (IU/L)	58.0 (31.0–107.0)	54.0 (31.5–106.5)	58.0 (31.2–109.0)	0.76
Total bilirubin (mg/dL)	0.9 (0.7–1.5)	1.1 (0.8–1.4)	0.9 (0.7–1.6)	0.53
Platelets (×10^9^/mL)	220.0 (164.0–268.0)	106.0 (72.0–148.0)	230.5 (185.8–270.2)	< 0.01
FIB‐4	1.1 (0.8–2.1)	4.2 (3.0–6.3)	1.0 (0.7–1.6)	< 0.01
< 1.3^b^	166 (57.5)	2 (4.9)	164 (66.1)	< 0.01
1.3^b^–2.67	72 (24.9)	8 (19.5)	64 (25.8)	
> 2.67	51 (17.6)	31 (75.6)	20 (8.1)	
ELF	9.1 (8.4–10.1)	11.4 (10.7–12.0)	9.0 (8.3–9.5)	< 0.01
< 9.8	202 (69.9)	2 (4.9)	200 (80.6)	< 0.01
≥ 9.8 to < 11.3	54 (18.7)	15 (36.5)	39 (15.8)	
≥ 11.3	33 (11.4)	24 (58.6)	9 (3.6)	
LSM (kPa)	7.8 (6.1–11.4)	21.0 (14.8–29.6)	7.5 (5.8–9.2)	< 0.01
< 10	202 (69.9)	4 (9.7)	198 (79.8)	< 0.01
10–15	41 (14.2)	7 (17.1)	34 (13.7)	
> 15	46 (15.9)	30 (73.2)	16 (6.5)	
*Histology*				
F0‐1‐2	207 (71.6)	6 (14.7)	201 (81.0)	< 0.01
F3	46 (15.9)	8 (19.5)	38 (15.3)	
F4	36 (12.5)	27 (65.8)	9 (3.4)	
*FIB4*– > *LSM* ^a^				
Low risk	198 (68.5)	3 (7.4)	195 (78.6)	< 0.01
Intermediate risk	21 (7.3)	1 (2.4)	20 (8.1)	
High risk	70 (24.2)	37 (90.2)	33 (13.3)	

*Note:* Data expressed as median(1st‐3rd quartiles) for continuous variables, number (percentage) for categorical variables.

Abbreviations: ALT, alanine aminotransferase; AST, aspartate aminotransferase; BMI, Body Mass Index; ELF, enhanced liver fibrosis test; FIB‐4, fibrosis‐4 index; GGT, gamma glutamyltransferase; LSM, liver stiffness measurement.

^a^ Two‐step approach: Low‐risk patients were patients with FIB‐4 < 1.3 (or 2.0 if age ≥ 65 years) or FIB‐4 1.3 [2 in > 65 years]–2.67 but LSM < 8 kPa, while high‐risk patients were those with FIB‐4 > 2.67 or FIB‐4 1.3 [2 in > 65 years]–2.67 and LSM > 12 kPa; the remaining patients were classified as intermediate risk.

^b^ 2 for patients older than 65 years.

* *p* value for difference between groups by Wilcoxon rank sum test or Fischer's exact test.

At histopathological assessment, 46 patients (15.9%) had advanced fibrosis (F3) and 36 (12.4%) had cirrhosis (F4) (Table 1). Agreement between the two liver pathologists was high for both MASH diagnosis (Cohen's *κ* = 0.80) and fibrosis staging (κ = 0.86). Among patients with cirrhosis at baseline, median ELF, LSM, and FIB4 values were 11.65 (IQR 10.8–12.32), 24.9 kPa (IQR 18.75–34.72 kPa) and 4.47 (IQR 2.87–6.34), respectively.

### 3.2 Liver Related Events

During a median follow‐up of 64 months (IQR 39–81), 41 patients (14.2%) experienced the composite primary endpoint, the majority of whom (27) had cirrhosis (F4) at baseline. Ascites, HCC, variceal bleeding and ascites were the most frequent liver‐related events, occurring in 13, 11, and 10 patients, respectively (Table S2). Patients who developed the composite endpoint were older (age 64 vs. 47), had a higher prevalence of type 2 diabetes (61.0% vs. 22.6%), and a lower BMI at baseline (27.3 vs. 28.7). Baseline values of FIB4, ELF and LSM were higher in patients who developed the composite endpoint (Table 1). AD occurred in 21 patients (7.2%), while ACLF occurred in 16 patients (5.5%) (Table S2).

### 3.3 Sample Size Considerations and Statistical Power

Given the observational design of the study, no a priori sample‐size calculation was performed. Instead, we evaluated the detectable effect size for time‐to‐event comparisons across ELF strata based on the observed number of outcome events, using Schoenfeld's approximation for Cox proportional hazards models. With 41 liver‐related events and an unbalanced distribution across extreme ELF categories (ELF < 9.8: *n* = 202; ELF ≥ 11.3: *n* = 33), assuming a two‐sided *α* = 0.05, the minimum detectable hazard ratio for a pairwise Cox comparison at 80% power is approximately HR ≈3.5. Accordingly, the study is powered to detect large prognostic effects between extreme ELF strata, whereas smaller effects may remain undetected. For this reason, inference focuses on effect sizes and 95% confidence intervals, and ELF was primarily analysed as a continuous variable to maximize statistical power.

### 3.4 Prognostic Role of NITs for LRE

**FIGURE 1:**
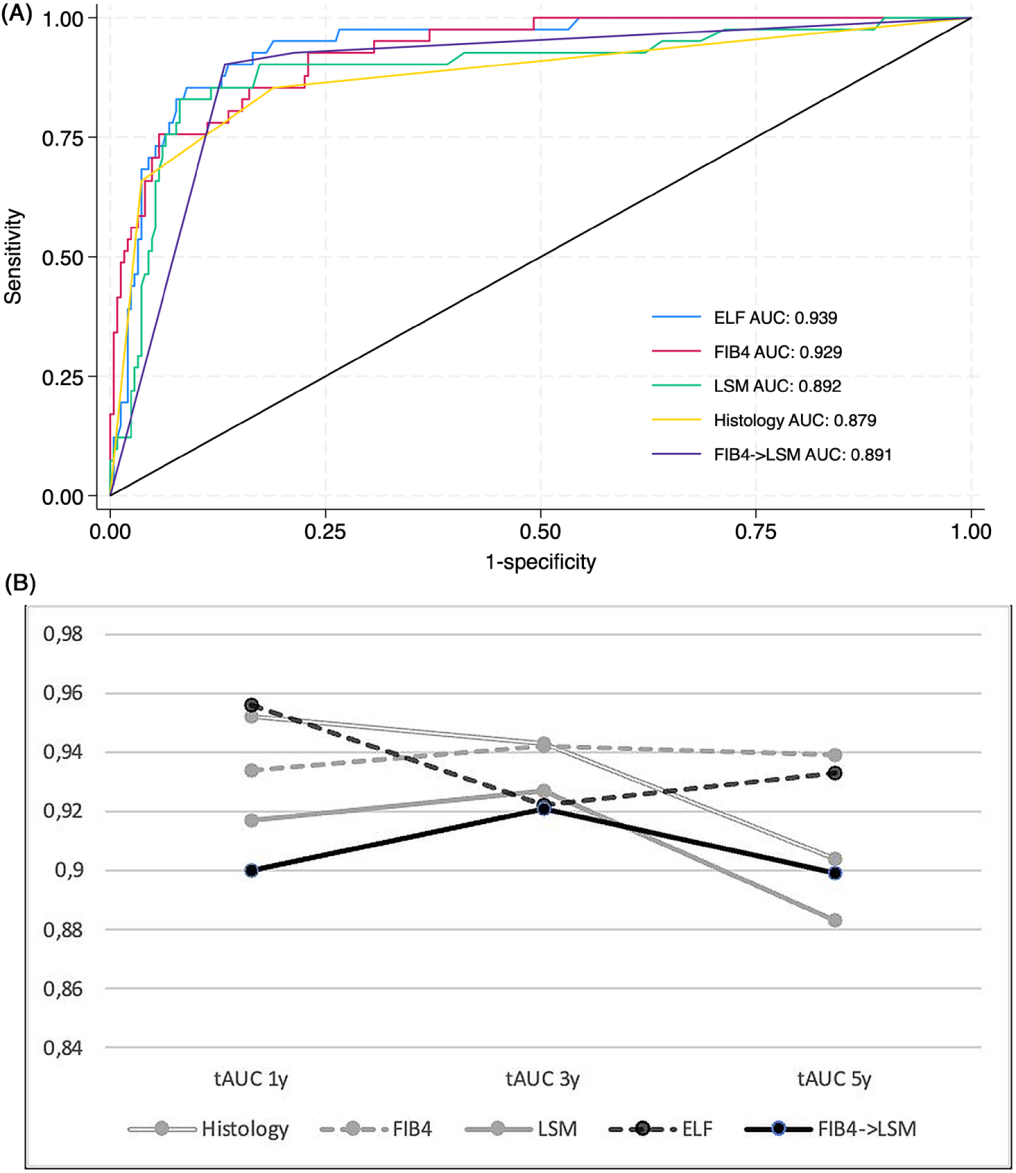
Overall and Time‐Dependent AUCs for Prediction of Composite Outcome. (A) Overall AUCs. Bar chart comparing the overall area under the curve (AUC) for predicting the composite outcome using liver fibrosis stage at histology, the Enhanced Liver Fibrosis (ELF) test, FIB‐4, Liver Stiffness Measurement (LSM) and the FIB‐4 + LSM two‐step approach. (B) Time‐Dependent AUCs. Line graph showing the time‐dependent AUCs for predicting the composite outcome at 1, 3 and 5 years for the same five predictive models (liver fibrosis stage at histology, ELF, FIB‐4, LSM and the FIB‐4 + LSM two‐step approach).

The discriminatory ability of NITs, including FIB‐4, ELF and LSM, for prediction of the composite primary endpoint was overall comparable to that of histological liver fibrosis stage. In overall analyses, no statistically significant differences versus histology were detected by DeLong's test for FIB‐4, LSM or the two‐step FIB‐4 → LSM approach, whereas ELF showed a modestly higher overall AUC compared with histology (AUC 0.939 vs. 0.879; *p* = 0.035) (Table 2). Therefore, ELF demonstrated the highest overall discrimination for the composite endpoint (AUC 0.939, 95% CI 0.905–0.973) among the evaluated tests, followed by FIB‐4 (AUC 0.929, 95% CI 0.890–0.967), LSM (AUC 0.892, 95% CI 0.830–0.955) and histological liver fibrosis stage (AUC 0.879, 95% CI 0.815–0.943) (Figure 1). In time‐dependent analyses at 1, 3 and 5 years, FIB‐4, ELF and LSM showed discriminative performance similar to histological fibrosis stage, with no statistically significant differences after correction for multiple comparisons at most time points (Table 2, Figure 1). A statistically significant difference was observed only for the two‐step FIB‐4 → LSM approach at 1 year, whereas no significant differences were detected at 3 or 5 years.

**TABLE 2:** Overall and time dependent AUCs for prediction of composite outcome by ELF, FIB4, LSM and liver fibrosis stage at histology.

	Overall AUC	*p* *	tAUC 1 year	*p* *	tAUC 3 years	*p* *	tAUC 5 years	*p* *
Histology	0.879 (0.815–0.943)	Ref	0.952 (0.928–0.975)	Ref	0.943 (0.900–0.986)	Ref	0.904 (0.842–0.965)	Ref
FIB4	0.929 (0.890–0.967)	0.078	0.934 (0.888–0.981)	0.6285	0.942 (0.908–0.975)	0.97	0.939 (0.901–0.977)	0.4
LSM	0.892 (0.830–0.955)	0.597	0.917 (0.857–0.976)	0.47 216	0.927 (0.891–0.964)	0.67	0.883 (0.812–0.953)	0.4
ELF	0.939 (0.905–0.973)	0.035	0.956 (0.930–0.982)	0.77851	0.922 (0.877–0.968)	0.67	0.933 (0.895–0.971)	0.4
FIB4‐ > LSM^a^	0.891 (0.843–0.940)	0.626	0.900 (0.876–0.923)	0.00021	0.921 (0.899–0.943)	0.67	0.899 (0.852–0.945)	0.83

*Note:* 95% confidence interval in brackets.

* *p* value for DeLong test. *p* values are adjusted for multiple testing (Benjamini–Hochberg).

^a^ Two‐step approach: Low‐risk patients were patients with FIB‐4 < 1.3 (or 2.0 if age ≥ 65 years) or FIB‐4 1.3 [2 in > 65 years]–2.67 but LSM < 8 kPa, while high‐risk patients were those with FIB‐4 > 2.67 or FIB‐4 1.3 [2 in > 65 years]–2.67 and LSM > 12 kPa; the remaining patients were classified as intermediate risk.

**FIGURE 2:**
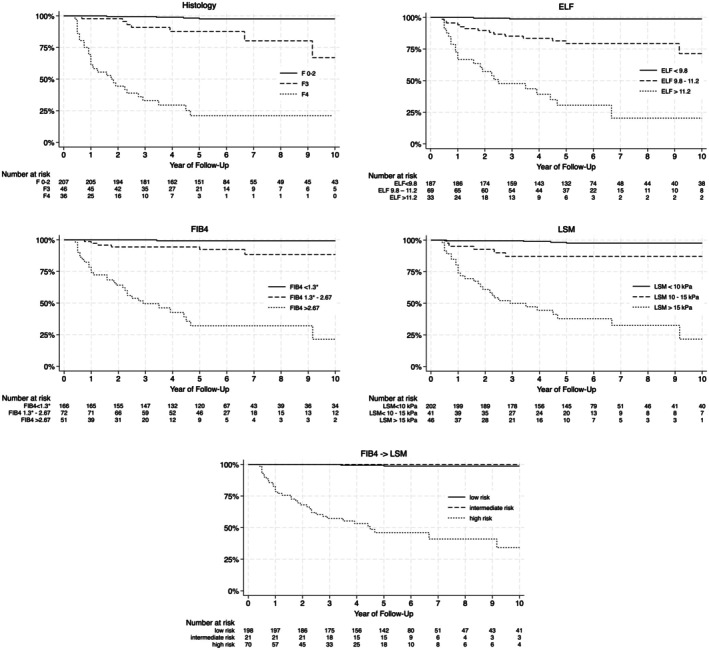
Kaplan–Meier Curves for Time to Primary Outcome. Kaplan–Meier curves illustrating the cumulative incidence of the primary composite outcome over time. Patients were stratified into low, intermediate and high‐risk groups based on pre‐defined cutoffs for the following indices: *Histology*: Low (F0‐2), Intermediate (F3), High (F4); *ELF*: Low (< 9.8), Intermediate (≥ 9.8 to < 11.3), High (≥ 11.3); *FIB‐4*: Low (< 1.3, or < 2.0 if age > 65 years), Intermediate (1.3 [or 2.0 if > 65] to 2.67), High (> 2.67); LSM: Low (< 10 kPa), Intermediate (10–15 kPa), High (> 15 kPa); Two‐Step Approach: Low‐risk patients (FIB‐4 < 1.3 [or 2.0 if age ≥ 65] OR FIB‐4 1.3–2.67 [or 2.0–2.67 if ≥ 65] with LSM < 8 kPa), Intermediate‐risk patients (remaining patients), High‐risk patients (FIB‐4 > 2.67 OR FIB‐4 1.3–2.67 [or 2.0–2.67 if ≥ 65] with LSM > 12 kPa).

Patients were stratified into three risk categories according to pre‐defined cut‐offs for ELF (< 9.8, ≥ 9.8 to < 11.3, ≥ 11.3), FIB‐4 (< 1.3 [< 2.0 in patients aged > 65 years], 1.3 [≥ 2.0 in patients aged ≥ 65 years] to < 2.67, ≥ 2.67), LSM (< 10, 10–15, > 15 kPa), histological fibrosis stage (F0‐2, F3, F4), and the two‐step FIB4 → LSM approach. Kaplan–Meier analyses showed statistically significant separation of survival curves across low, intermediate and high‐risk categories for all evaluated stratification methods (log‐rank *p* < 0.05; Figure 2). Consistently, univariable Cox regression analyses demonstrated progressively increasing hazards of the composite outcome across risk strata defined by ELF, LSM, FIB‐4, histological fibrosis stage and the two‐step approach (Table 3).

**TABLE 3:** Risk of primary composite outcome for 3 risk groups defined by test‐specific cut‐offs.

	Events/patients in group (%)	Hazard ratio (95% CI)	*p*
*ELF*			
< 9.8	2/202 (1%)	1 (Reference)	
≥ 9.8 to < 11.3	15/54 (27.8%)	35.39 (8.07–155.31)	< 0.001
≥ 11.3	24/33 (72.7%)	140.51 (32.90–600.15)	< 0.001
*LSM*			
< 10 kPa	4/202 (2%)	1 (Reference)	
10–15 kPa	7/41 (17.1%)	9.32 (2.73–31.88)	< 0.001
> 15 kPa	30/46 (65.2%)	55.95 (19.49–160.62)	< 0.001
*Histology*			
F0‐1‐2	6/207 (2.9%)	1 (Reference)	
F3	8/46 (17.4%)	7.66 (2.63–22.25)	< 0.001
F4	27/36 (75%)	78.02 (29.64–205.42)	< 0.001
*FIB‐4*			
< 1.3^a^	2/166 (1.2%)	1 (Reference)	
1.3^a^–2.67	8/72 (11.1%)	9.53 (2.02–44.92)	0.004
> 2.67	31/51 (60.8%)	93.68 (22.23–394.73)	< 0.001
*FIB‐4 → LSM* ^b^			
Low risk	3/198 (1.5%)	1 (Reference)	
Intermediate risk	1/21 (4.8%)	3.07 (0.32–29.59)	0.33
High risk	37/70 (52.9%)	55.37 (16.99–180.46)	< 0.001

*Note:* Hazard ratios from univariable Cox regression for prediction of composite endpoint according to 3 groups of low, intermediate and high risk, with *p* values for between‐group.

^a^ 2 for patients older than 65 years.

^b^ Two‐step approach: Low‐risk patients were patients with FIB‐4 < 1.3 (or 2.0 if age ≥ 65 years) or FIB‐4 1.3 [2 in > 65 years]–2.67 but LSM < 8 kPa, while high‐risk patients were those with FIB‐4 > 2.67 or FIB‐4 1.3 [2 in > 65 years]–2.67 and LSM > 12 kPa; the remaining patients were classified as intermediate risk.

In particular, compared with patients with ELF < 9.8, those with ELF ≥ 9.8 and < 11.3 and those with ELF ≥ 11.3 showed markedly higher risks of the composite outcome, with hazard ratios of 35.4 (95% CI 8.1–155.3) and 140.5 (95% CI 32.9–600.1), respectively (Table 3). Given the limited number of events, these estimates should be interpreted as indicators of strong risk gradients rather than precise effect sizes.

In sensitivity analyses using multivariable Cox regression models adjusted for age, sex, body mass index and type 2 diabetes, higher risk categories defined by ELF remained consistently associated with an increased risk of liver‐related events compared with the low‐risk group. In particular, both ELF ≥ 9.8 and ELF ≥ 11.3 were associated with markedly higher hazards of the composite outcome relative to ELF < 9.8 after adjustment for clinical covariates (adjusted HR 26.15, 95% CI 5.38–127.21 and adjusted HR 105.61, 95% CI 20.48–544.56, respectively; Table S3).

Similarly, intermediate‐ and high‐risk categories defined by histological fibrosis stage showed higher adjusted risks of development of composite outcome compared with patients with F0‐2 fibrosis (F3: adjusted HR 6.44, 95% CI 1.88–22.03; F4: adjusted HR 38.46, 95% CI 11.63–127.23). LSM and FIB‐4 showed comparable performance. Both intermediate‐ and high‐risk categories remained significantly associated with increased risk after multivariable adjustment, with the magnitude of effect ~5 (adjusted HR) but wider confidence intervals for FIB‐4 (Table S3).

In the two‐step FIB‐4 → LSM approach, the intermediate‐risk group did not show a significantly increased adjusted risk compared with the low‐risk group (adjusted HR 1.93, 95% CI 0.20–19.06), whereas the high‐risk group accounted for the majority of liver‐related events (37 of 41 events) and remained strongly associated with the composite outcome after adjustment (adjusted HR 27.31, 95% CI 7.66–97.33; Table S3). Incidence rates of the composite outcome increased markedly across ELF‐based risk groups, from 0.14 per 100 person‐years in the low‐risk group (ELF < 9.8) to 5.35 per 100 person‐years in the intermediate‐risk group (ELF 9.8– < 11.3) and 23.4 per 100 person‐years in the high‐risk group (ELF ≥ 11.3) (Table 4). When compared with the low‐risk reference category, IRRs indicated substantially higher event rates in both the intermediate‐ and high‐risk ELF groups, with IRRs of 39.0 and 170.6, respectively (Table 4). Similar stepwise increases in incidence rates were observed across histology‐based risk categories (Table 4).

**TABLE 4:** Incidence rate of composite outcome per 100 person‐years and incidence rate ratio head‐to‐head comparison stratified by ELF, LSM, FIB and histology according to pre‐defined cutoffs.

Group	Events/PY	Incidence rate (per 100 PY)	Incidence rate ratio vs low risk
*ELF*			
< 9.8	2/1456.2	0.14	1 (Reference)
9.8–< 11.3	15/280.3	5.35	39
≥ 11.3	24/102.4	23.43	170.6
*LSM*			
< 10	4/1414.2	0.28	1 (Reference)
10–< 15	7/262.5	2.67	9.4
≥ 15	30/162.2	18.5	65.4
*Histology*			
F0–F2	6/1491	0.4	1 (Reference)
F3	8/263	3.04	7.6
F4	27/84.9	31.8	79
*FIB4*			
Low	2/1180.8	0.17	1 (Reference)
Intermediate	8/491	1.63	9.6
High	31/167.2	18.54	109.5
*FIB‐4 → LSM* ^a^			
Low	3/1413	0.21	1 (Reference)
Intermediate	1/151.9	0.66	3.1
High	37/274	13.5	63.6

*Note:* For each non‐invasive test is showed the incidence rate per 100 person‐years (PY) and the incidence rate ratio (IRR) between intermediate and high‐risk group versus low‐risk group. Risk groups are defined by test‐specific cut‐offs.

^a^ Two‐step approach: Low‐risk patients were patients with FIB‐4 < 1.3 (or 2.0 if age ≥ 65 years) or FIB‐4 1.3 [2 in > 65 years] – 2.67 but LSM < 8 kPa, while high‐risk patients were those with FIB‐4 > 2.67 or FIB‐4 1.3 [2 in > 65 years] – 2.67 and LSM > 12 kPa; the remaining patients were classified as intermediate risk.

After weighting by the inverse probability of treatment to account for differences in age and sex across ELF categories, the estimated potential outcome means for liver‐related events showed a clear gradient across ELF strata. In the low ELF group, the IPTW‐adjusted probability of liver‐related events was 1.2% (95% CI –0.4% to 3.0%). This probability increased to 18.9% (95% CI 10.2%–27.5%) in the intermediate ELF group and to 62.8% (95% CI 33.2%–92.5%) in the high ELF group. These IPTW‐weighted estimates indicate progressively higher even probabilities across increasing ELF categories, consistent with the unweighted analyses and supporting the presence of a strong risk gradient (Table S4).

With regard to secondary outcomes, Kaplan–Meier analyses showed significant separation of survival curves across low‐, intermediate‐ and high‐risk categories defined by ELF, LSM, FIB4 and histological liver fibrosis stage for both AD and ACLD development (log‐rank *p* < 0.05; Figures S1 and S2). Consistently, univariable Cox regression analyses demonstrated progressively increasing hazards of secondary outcomes across risk strata defined by ELF categories (Table S5). Given the limited number of secondary outcome events, hazard ratios are reported as descriptive indicators of risk gradients rather than as precise effect estimates.

## 4 Discussion

In this longitudinal study of 289 biopsy‐proven MASLD patients followed for a median follow‐up of 64 months, we demonstrate that the Enhanced Liver Fibrosis (ELF) test provides prognostic stratification for liver‐related events (LRE) that is comparable to histologically assessed fibrosis stage. Across multiple analytical approaches, higher ELF values were consistently associated with substantially increased risks of LREs, supporting the clinical relevance of ELF as a non‐invasive prognostic tool in MASLD.

Patients with intermediate (ELF ≥ 9.8 to < 11.3) and high (ELF ≥ 11.3) risk categories showed markedly higher hazards of LREs compared with those in the low‐risk group (ELF < 9.8), even after adjustment for age, sex, body mass index and type 2 diabetes. While the magnitude of these hazard ratios was large, the associated confidence intervals were wide, reflecting the limited number of outcome events and the resulting statistical imprecision. These estimates should therefore be interpreted primarily as indicators of strong risk gradients rather than as precise effect sizes.

Liver fibrosis consists in the progressive deposition of extracellular matrix (ECM) proteins, mostly type I and III collagen, which can alter the physiological architecture of the liver [30]. The excessive accumulation of ECM, together with dysregulated anti‐fibrotic processes, leads to extensive replacement of liver parenchyma by fibrotic tissue and, eventually, a loss of liver function [31]. The ELF assay is based on the direct measurement of circulating markers of hepatic ECM metabolism, namely hyaluronic acid (HA), tissue inhibitor of metalloproteinases‐1 (TIMP‐1) and propeptide of type III procollagen (PIIINP). More specifically, TIMP‐1 is a strong inhibitor of the family of matrix metalloproteinases (MMPs) and is involved in matrix remodelling [32]. HA is a critical component of ECM and PIIINP derives both from the de novo synthesis of collagen and the turnover of pre‐existing fibres [33, 34]. The ELF test offers several advantages, including ease of sample collection, automated analysis, and reproducibility of results. Thus, ELF has been shown to be an excellent non‐invasive predictor of liver fibrosis with strong diagnostic and prognostic performance across many different liver disease etiologies [35, 36]. Several studies have examined the diagnostic utility of ELF testing in relationship to fibrosis staging in patients with MASLD. As a result, this test can be used alone or in combination with other NITs, such as FIB‐4, to identify or exclude advanced liver fibrosis [37, 38]. In secondary care settings, ELF has shown good accuracy for the diagnosis of advanced fibrosis and cirrhosis in patients with MASLD [39, 40]. Siemens has recommended two pre‐defined thresholds for prognostic use: 9.8 and 11.3. In a large metanalysis, Vali et al. [41] demonstrated that the low threshold (7.7) rules out significant fibrosis with high sensitivity and NPV in both low‐ and high‐prevalence settings. On the other hand, the upper threshold (9.8) exhibited good specificity for ruling in advanced fibrosis (0.86) but PPV was only satisfactory (> 0.7) in high prevalence settings (> 40%) [41]. Higher thresholds (11.3 validated in a large cohort [42]) showed a better sensitivity and PPV for diagnosis of advanced fibrosis and liver cirrhosis respectively, particularly in secondary and tertiary care settings.

A recent individual participant data meta‐analysis revealed that non‐invasive tests, including FIB4 and LSM (but not ELF), have excellent prognostic ability comparable to histologically assessed liver fibrosis in MASLD patients [5]. In several other studies ELF test has been investigated and validated as a prognostic factor in MASLD patients [18, 19] in comparison with other NITs but in the absence of histopathological assessment. Are et al. [18] demonstrated ELF prognostic ability in a cohort of 161 patients with MASH and compensated cirrhosis within a clinical trial setting (NCT02462967), with short‐term outcomes assessed over a 52‐week period. Johnson et al. [19] evaluated ELF prognostic ability in a cohort of 243 patients with a long median follow‐up of 50 months, but without comparison to histopathological data. Pearson et al. [38] recently confirmed ELF prognostic ability in a large cohort of 1327 patients; moreover, the study was not histologically controlled, and this limits its accuracy in correlating ELF with true fibrosis stages. Younossi et al. [20] showed that ELF is a good predictor of adverse clinical outcomes in a large population of 2154 patients with MASH and fibrosis stage F3 or 4 who were enrolled in four Phase 2 or 3 clinical trials, with a median follow‐up of 16 months. In contrast to previous studies, our analysis was conducted in a real‐life clinical setting where all patients had undergone liver biopsy. This unique feature allowed us to directly compare the prognostic performance of ELF with histologically defined fibrosis. Moreover, while the trial‐derived cohort analysed by Younossi et al. [20] may be subjected to selection bias and was followed for a relatively short median period of 16 months, our cohort had a substantially longer median follow‐up of 64 months and a higher proportion of liver‐related events. These characteristics provide greater real‐world clinical relevance to our findings and strengthen the evidence supporting the prognostic value of ELF.

The overall incidence of LRE in our study was slightly higher than that reported in prior studies with comparable median follow‐up durations [5, 19]. In our study, 14.2% of patients developed LRE compared with 5.8% and 7.8% reported by Mozes et al. [5] and Johnson et al. [19], respectively. Although the prevalence of advanced fibrosis (*F* > 2) was similar (28.4% in our study vs. 32.6% in Mozes et al. [5] study), patients who developed LRE in our cohort had higher baseline NITs compared to patients who developed events in other cohorts. Indeed, the median baseline LSM value of patients who developed LRE was 21.0 kPa in our study vs. 14.8 kPa and 17.1 kPa in the already cited studies by Mozes et al. [5] and Johnson et al. [19]. In the study by Younossi et al. [20], 7% of patients with F3 and 7.3% of patients with F4 developed LRE. In contrast, in our cohort, 42.7% of patients with advanced fibrosis (F3–F4) experienced LRE, including 17.4% of patients with F3 and 75.0% of those with F4 fibrosis. However, the median follow up period in that study (16 months) was significantly lower than that of our study (64 months). Overall, the higher incidence of LRE in our study is likely due to the inclusion of a high rate of patients with more severe liver disease, as evidenced by higher LSM values at baseline, as well as to the longer follow‐up period, which allows for the observation of more events. This distribution suggests that the overall analysis is possibly driven by complications arising from pre‐existing cirrhosis, rather than reflecting true disease progression from earlier fibrosis stages. Additionally, differences in cohort selection criteria across studies contribute to the variation in reported LRE rates. Given the limited number of events, comparative analyses between NITs should be interpreted cautiously and primarily as exploratory.

The prognostic stratification established by liver biopsy, LSM and FIB4 shown in our study supports the findings already highlighted in the literature [5, 18, 19, 20, 43]. In particular, it confirms that patients in the low‐risk categories (F0‐2, LSM < 10 kPa or FIB4 < 1.3) have a negligible risk of developing LREs. As fibrosis progresses, both the risk category and the probability of LREs increase. This pattern is particularly evident for histology‐ and LSM‐based risk assessment, while FIB4, in our study, does not appear to effectively differentiate the risk of developing events within intermediate‐ and low‐risk classes.

The prognostic stratification ability of ELF is comparable to that of liver biopsy and LSM in our study. The magnitude of HRs should be interpreted cautiously due to sparse events in extreme ELF strata. Among patients who developed LRE, only 4.8% had ELF < 9.8 confirming the negligible risk of worsening liver disease for the low‐risk class, and this finding contrasts with what has been reported by Johnson et al. [19] who showed that 26.3% of patients developing events had ELF < 9.8. This disparity may be attributed to differences between the two cohorts. The patients included in the Johnson et al. [19] study were selected as at‐risk MASH patients from a diabetes clinic setting and therefore had a higher burden of comorbidities (at baseline, 10% of patients had extrahepatic malignancies, 22% had cardiovascular disease and diabetes prevalence was 83% vs. 28% of our cohort). Thus, the burden of comorbidities may impact prognosis and event risk alongside liver fibrosis. Indeed, in other studies involving MASLD patients [43] or general population cohorts [12], ELF < 9.8 showed a high negative predictive value (> 98%) for decompensation events. In line with the most recent EASL guidelines [22], we also applied a multi‐layer approach combining FIB‐4 and LSM to stratify patients according to risk categories. In our cohort, the prognostic performance of this strategy was comparable to that of histology, LSM and ELF. Of note, given the limited sample size, the intermediate‐risk group did not differ from the low‐risk group in terms of liver‐related events, while almost all events (37 out of 41) occurred in patients classified as high risk. This finding suggests that, although the multi‐layer strategy may have utility in identifying patients at highest risk, its discriminative power across the lower risk strata might be limited, even if these results should be assessed in cohorts larger than the present one.

The main strength of our study is the head‐to‐head comparison of ELF with other widely used NITs (FIB4 and LSM) and the traditional gold standard of liver biopsy. For every NIT, we evaluated well‐established and validated cut‐off points, avoiding overly optimistic prognostic accuracies derived from population‐specific thresholds. Moreover, the use of already validated cut‐offs facilitates the integration of our findings into routine clinical practice.

Although ELF showed higher AUCs than other non‐invasive tests, these differences were not statistically significant, reflecting the limited sample size and number of events. Therefore, our findings should be interpreted as evidence of non‐inferiority rather than clear superiority, supporting the potential role of ELF as a clinically valuable and less invasive alternative to histological assessment, while acknowledging the need for larger studies to confirm whether significant differences exist among available tests.

Another strength of our study lies in the event assessment methodology. Events were identified not only through a manual review of electronic health records but also via follow‐up calls and short visits for patients lost to follow‐up. This approach allowed us to obtain a reliable and extended median follow‐up period, which is crucial for studying prognosis in a patient population where decompensation events develop slowly and are relatively infrequent.

Nevertheless, several limitations warrant consideration. First, the retrospective, single‐centre design and the inclusion of only subjects with histologically proven MASLD limit the generalisability of our findings. This limitation is further compounded by the fact that our cohort consists exclusively of patients who underwent liver biopsy for clinical indications, thus likely representing a more advanced disease spectrum than that seen in the general MASLD population. Another limitation is that we did not repeat the noninvasive tests (NITs) over time, so we could not track how fibrosis progresses or regresses in individual patients. Capturing those dynamic changes would have added prognostic value beyond a single baseline measurement. In real‐world clinical practice, patients are usually monitored regularly, but our retrospective design made it difficult to ensure that NITs were measured at consistent time points for everyone. This was especially challenging for the ELF score, which we had to measure from stored serum samples. Another limitation is that, because this was a retrospective study, the serum samples used for ELF testing had been stored at −80°C. While hyaluronic acid (HA) is generally considered stable under those conditions, we cannot completely rule out some degradation or aggregation over time—which might introduce variability in the HA component and affect the reproducibility of the ELF score compared to testing fresh samples prospectively. That said, published evidence does suggest that HA, PIIINP and TIMP‐1 remain acceptably stable even after long‐term storage, and any impact on the ELF score is unlikely to be clinically meaningful [44]. On top of that, the ELF test is a commercial assay that comes with added costs, and reimbursement policies in some regions may limit its use—which could make it harder to apply in everyday clinical practice.

In conclusion, our study reinforces the role of non‐invasive tests as reliable prognostic markers in MASLD. Our findings demonstrate that the ELF test and LSM, when using widely accepted cut‐off values, can identify MASLD patients at risk of future liver‐related decompensation events and liver‐related mortality, performing comparably to liver biopsy. Nonetheless, further research with larger groups of patients, including those who have not had a biopsy, with repeated NITs over time and longer follow‐up periods is needed. This will help fine‐tune predictive models to better guide patient monitoring and treatment decisions.

## Author Contributions

Conception or design of the work: Antonio Liguori and Luca Miele. Data collection: Antonio Liguori, Francesca D'Ambrosio, Consuelo Cefalo, Simone Galletti, Sara Cardinali, Maria Cristina Giustiniani and FPG‐UCSC PROMETEO—ELF substudy Research Group. Data analysis and interpretation: Antonio Liguori, Giuseppe Marrone, Marco Biolato, Luca Miele and Tommaso Mazza. Drafting the article: Antonio Liguori, Francesca D'Ambrosio and Giulia Angelini. Critical revision of the article: Giulia Angelini, Gianludovico Rapaccini, Maurizio Pompili, Antonio Grieco, Andrea Urbani, Maurizio Sanguinetti, Antonio Gasbarrini, Luca Miele and Tommaso Mazza. Final approval of the version to be published: all authors.

## Funding

This work was supported by Siemens‐Healthineers unrestricted grant (to L.M.). Facilities at CEMAD are supported by Fondazione Roma (FR CEMAD 2021–25).

## Ethics Statement

The local Ethical Committee approved the study (local ID 2576).

## Consent

Informed consent was obtained from all individuals included in this study.

## Conflicts of Interest

The authors declare no conflicts of interest.

## Supporting information


**Table S1:** TRIPOD checklist.
**Table S2:** The type of liver related events developed by patients.
**Table S3:** The association between index tests (ELF, FIB4, LSM and Histology) and liver related events using multivariable Cox regression models adjusted for age, sex, body mass index and type 2 diabetes.
**Table S4:** IPTW‐weighted estimates to account for potential confounding due to differences in baseline age and sex across ELF categories.
**Table S5:** Results of Cox regression models for prediction of secondary outcomes (acute decompensation or acute on chronic liver failure).

**Figure S1:** Kaplan–Meier curves for time to AD according to histology, FIB‐4, LSM and ELF pre‐defined cut‐offs. Kaplan–Meier curves for time to the development of ascites (AD). Patients were stratified into low, intermediate, and high‐risk groups based on pre‐defined cutoffs for ELF, FIB‐4, histology, and LSM. Risk group definitions: High risk: ELF > 11.2, FIB‐4 > 2.67, Histology F4, LSM > 15 kPa; Intermediate risk: ELF 9.8–11.2, FIB‐4 1.3–2.67 (or 2.0–2.67 if age > 65 years), Histology F3, LSM 10–15 kPa; Low risk: ELF < 9.8, FIB‐4 < 1.3 (or < 2.0 if age > 65 years), Histology F0‐2, LSM < 10 kPa. Time is expressed in years.

**Figure S2:** Kaplan–Meier Curves for Time to ACLF According to Histology, FIB‐4, LSM and ELF Pre‐defined Cutoffs. Kaplan–Meier curves for time to the development of acute‐on‐chronic liver failure (ACLF). Patients were stratified into low, intermediate, and high‐risk groups based on pre‐defined cutoffs for ELF, FIB‐4, histology, and LSM. Risk Group Definitions: High Risk: ELF > 11.2, FIB‐4 > 2.67, Histology F4, LSM > 15 kPa; Intermediate Risk: ELF 9.8–11.2, FIB‐4 1.3–2.67 (or 2.0–2.67 if age > 65 years), Histology F3, LSM 10–15 kPa; Low Risk: ELF < 9.8, FIB‐4 < 1.3 (or < 2.0 if age > 65 years), Histology F0‐2, LSM < 10 kPa. Time is expressed in years.

## Data Availability

Data are available on request due to privacy/ethical restrictions.
