# Supplementary material for: Enhanced Liver Fibrosis Test, FIB‐4 and FibroScan: Real‐World Prognostic Accuracy for MASLD in a Biopsy‐Controlled Cohort
Source: Liver Int. 2026 Jul 2;46(8):e70774. doi: 10.1111/liv.70774 (PMC13329087; doi:10.1111/liv.70774)
Supplement: Supplementary file 1 — Table S1: TRIPOD checklist. Table S2: The type of liver related events developed by patients. Table S3: The association between index tests (ELF, FIB4, LSM and Histology) and liver related events using multivariable Cox regression models adjusted for age, sex, body mass index and type 2 diabetes. Table S4: IPTW‐weighted estimates to account for potential confounding due to differences in baseline age and sex across ELF categories. Table S5: Results of Cox regression models for prediction of secondary outcomes (acute decompensation or acute on chronic liver failure). [file LIV-46-0-s002.docx]

SUPPLEMENTARY MATERIAL

Table S1: TRIPOD Checklist

| **Section/Topic** | **Item** | **Checklist Item** | **Page** |
| --- | --- | --- | --- |
| **Title and abstract** | | | |
| Title | 1 | Identify the study as developing and/or validating a multivariable prediction model, the target population, and the outcome to be predicted. | 1 |
| Abstract | 2 | Provide a summary of objectives, study design, setting, participants, sample size, predictors, outcome, statistical analysis, results, and conclusions. | 6-7 |
| **Introduction** | | | |
| Background and objectives | 3a | Explain the medical context (including whether diagnostic or prognostic) and rationale for developing or validating the multivariable prediction model, including references to existing models. | 9 |
|  | 3b | Specify the objectives, including whether the study describes the development or validation of the model or both. | 9-10 |
| **Methods** | | | |
| Source of data | 4a | Describe the study design or source of data (e.g., randomized trial, cohort, or registry data), separately for the development and validation data sets, if applicable. | 10-11 |
|  | 4b | Specify the key study dates, including start of accrual; end of accrual; and, if applicable, end of follow-up. | 10-11 |
| Participants | 5a | Specify key elements of the study setting (e.g., primary care, secondary care, general population) including number and location of centres. | 10-11 |
|  | 5b | Describe eligibility criteria for participants. | 10-11 |
|  | 5c | Give details of treatments received, if relevant. | Not applicable |
| Outcome | 6a | Clearly define the outcome that is predicted by the prediction model, including how and when assessed. | 10-12 |
|  | 6b | Report any actions to blind assessment of the outcome to be predicted. | 10-12 |
| Predictors | 7a | Clearly define all predictors used in developing or validating the multivariable prediction model, including how and when they were measured. | 10-13 |
|  | 7b | Report any actions to blind assessment of predictors for the outcome and other predictors. | 10-13 |
| Sample size | 8 | Explain how the study size was arrived at. | 9-13, 15 |
| Missing data | 9 | Describe how missing data were handled (e.g., complete-case analysis, single imputation, multiple imputation) with details of any imputation method. | 10-13 |
| Statistical analysis methods | 10a | Describe how predictors were handled in the analyses. | 10-13 |
|  | 10b | Specify type of model, all model-building procedures (including any predictor selection), and method for internal validation. | 10-13 |
|  | 10d | Specify all measures used to assess model performance and, if relevant, to compare multiple models. | 10-13 |
| Risk groups | 11 | Provide details on how risk groups were created, if done. | 10-13 |
| **Results** | | | |
| Participants | 13a | Describe the flow of participants through the study, including the number of participants with and without the outcome and, if applicable, a summary of the follow-up time. A diagram may be helpful. | 14-17 |
|  | 13b | Describe the characteristics of the participants (basic demographics, clinical features, available predictors), including the number of participants with missing data for predictors and outcome. | 14-17 |
| Model development | 14a | Specify the number of participants and outcome events in each analysis. | 14-17 |
|  | 14b | If done, report the unadjusted association between each candidate predictor and outcome. | 14-17 |
| Model specification | 15a | Present the full prediction model to allow predictions for individuals (i.e., all regression coefficients, and model intercept or baseline survival at a given time point). | 14-17 |
|  | 15b | Explain how to the use the prediction model. | 14-17 |
| Model performance | 16 | Report performance measures (with CIs) for the prediction model. | 14-17 |
| **Discussion** | | | |
| Limitations | 18 | Discuss any limitations of the study (such as nonrepresentative sample, few events per predictor, missing data). | 18-22 |
| Interpretation | 19b | Give an overall interpretation of the results, considering objectives, limitations, and results from similar studies, and other relevant evidence. | 18-22 |
| Implications | 20 | Discuss the potential clinical use of the model and implications for future research. | 18-22 |
| **Other information** | | | |
| Supplementary information | 21 | Provide information about the availability of supplementary resources, such as study protocol, Web calculator, and data sets. | Supplementary material |
| Funding | 22 | Give the source of funding and the role of the funders for the present study. | 5 |

Supplementary Table 2: the type of Liver Related Events developed by patients

| Patient# | Ascites | HE | HCC | Liver transplant | SBP | Variceal bleeding | MELD>15 | Death | AD/ACLF |
| --- | --- | --- | --- | --- | --- | --- | --- | --- | --- |
| 1 | X | X |  |  |  |  |  |  | AD |
| 2 |  |  | X | X |  |  |  |  |  |
| 3 | X | X | X |  |  |  |  |  | ACLF gr. 2 |
| 4 |  |  |  |  |  | X |  |  | AD |
| 5 |  |  | X |  |  |  |  | X | ACLF gr. 3 |
| 6 |  | X |  |  |  |  |  |  | ACLF gr. 1 |
| 7 | X |  |  |  |  |  |  |  | AD |
| 8 | X |  |  |  | X |  |  |  | AD |
| 9 | X |  |  |  |  |  |  |  | AD |
| 10 | X |  |  |  |  |  |  |  | ACLF gr. 2 |
| 11 |  |  |  | X |  |  | X |  | AD |
| 12 |  |  |  | X |  |  | X |  | ACLF gr. 1 |
| 13 |  |  | X |  |  |  |  |  | AD |
| 14 |  |  | X |  |  |  |  |  |  |
| 15 | X | X | X |  |  |  |  | X | ACLF gr. 3 |
| 16 |  |  |  |  |  |  |  | X | AD |
| 17 |  |  |  |  |  | X |  |  | AD |
| 18 |  |  |  |  |  | X |  |  | AD |
| 19 |  |  |  |  |  | X |  |  | AD |
| 20 |  |  |  |  |  | X |  | X | ACLF gr. 2 |
| 21 | X | X | X | X | X |  | X |  | ACLF gr. 1 |
| 22 |  |  |  |  |  | X |  | X | ACLF gr. 2 |
| 23 |  |  |  |  |  | X |  |  | AD |
| 24 | X |  | X | X |  |  |  | X | ACLF gr. 2 |
| 25 |  |  |  |  |  | X |  |  | AD |
| 26 |  |  | X |  |  |  |  |  | AD |
| 27 |  |  |  | X |  |  | X |  | ACLF gr. 1 |
| 28 |  |  | X | X |  |  |  |  |  |
| 29 |  | X |  |  |  |  |  |  | AD |
| 30 |  |  |  |  |  | X |  |  | ACLF gr. 1 |
| 31 |  |  | X |  |  |  |  |  |  |
| 32 |  |  |  |  |  |  | X |  | AD |
| 33 |  |  |  |  |  | X |  |  | ACLF gr. 1 |
| 34 |  |  |  |  |  |  | X |  | AD |
| 35 | X |  |  |  |  |  |  |  | AD |
| 36 |  | X |  |  |  | X |  |  | ACLF gr. 1 |
| 37 | X |  |  |  |  |  |  |  | AD |
| 38 | X |  |  |  |  |  |  |  | AD |
| 39 |  |  |  | X |  |  | X |  | ACLF gr. 2 |
| 40 | X | X |  |  |  |  |  |  | ACLF gr. 1 |
| 41 |  | X |  |  |  |  |  |  | AD |

HE: Hepatic Encephalopathy, HCC: Hepatocellular carcinoma, SBP: Spontaneous bacterial peritonitis, AD: Acute Decompensation, ACLF: Acute-on-Chronic Liver Failure, gr.: grade

Supplementary Table 3: the association between index tests (ELF, FIB4, LSM, Histology) and liver related events using multivariable Cox regression models adjusted for age, sex, body mass index, and type 2 diabetes

|  | Adjusted Hazard Ratio (95% CI) | P value |
| --- | --- | --- |
| **ELF** |  |  |
| <9.8 | 1 (Reference) |  |
| ≥9.8 to <11.3 | 26.15 (5.38–127.21) | <0.001 |
| ≥11.3 | 105.61 (20.48–544.56) | <0.001 |
| **LSM** |  |  |
| <10 kPa | 1 (Reference) |  |
| 10-15 kPa | 5.22 (1.41–19.35) | 0.01 |
| >15 kPa | 24.20 (7.58–77.27) | <0.001 |
| **Histology** |  |  |
| F0-1-2 | 1 (Reference) |  |
| F3 | 6.44 (1.88–22.03) | 0.003 |
| F4 | 38.46 (11.63–127.23) | <0.001 |
| **FIB-4** |  |  |
| <1.3^†^ | 1 (Reference) |  |
| 1.3^†^-2.67 | 5.95 (1.23–28.77) | 0.027 |
| >2.67 | 35.40 (7.58–165.29) | <0.001 |
| **FIB-4 → LSM** |  |  |
| Low | 1 (Reference) |  |
| Intermediate | 1.93 (0.20–19.06) | 0,57 |
| High | 27.31 (7.66–97.33) | <0.001 |

Hazard ratios from univariable and multivariable Cox regression for prediction of secondary composite outcomes according to 3 groups of low, intermediate and high risk, with p values for between-group. Covariates of multivariable Cox regression are diabetes, age, gender Diabetes and BMI. †2 for patients older than 65 years

Supplementary Table 4: IPTW-weighted estimates to account for potential confounding due to differences in baseline age and sex across ELF categories

| **ELF group** | **Adjusted probability of LRE (POmean)** | **95% CI** | **Difference vs ELF <9.8 (ATE)** | **95% CI** | **p-value** |
| --- | --- | --- | --- | --- | --- |
| ELF <9.8 | 1.2% | –0.4% to 3.0% | Reference | – | – |
| ELF 9.8-11.2 | 18.9% | 10.2% to 27.5% | +17.6% | 8.8% to 26.4% | <0.001 |
| ELF >11.2 | 62.8% | 33.2% to 92.5% | +61.6% | 31.9% to 91.3% | <0.001 |

Adjusted probabilities of liver-related events (potential outcome means, POmeans) across ELF categories after inverse probability of treatment weighting (IPTW) to account for age and sex. Differences (average treatment effects, ATE) are shown relative to the low ELF group (reference). Robust variance estimators were used to calculate 95% confidence intervals

Supplementary Table 5: Results of Cox regression models for prediction of secondary outcomes (Acute Decompensation or Acute on Chronic Liver Failure)

|  | Hazard Ratio (95% CI) | P value | Adjusted Hazard Ratio (95% CI) | P value |
| --- | --- | --- | --- | --- |
| **ELF** |  |  |  |  |
| <9.8 | 1 (Reference) |  | 1 (Reference) |  |
| ≥9.8 to <11.3 | 19.08 (4.26 – 85.40) | <0.001 | 14.49 (3.10 – 67.73) | 0.001 |
| ≥11.3 | 118.06 (27.62 – 504.64) | <0.001 | 71.93 (14.58 –354.78) | <0.001 |
| **LSM** |  |  |  |  |
| <10 kPa | 1 (Reference) |  | 1 (Reference) |  |
| 10-15 kPa | 9.53 (2.78 – 32.57) | 0.002 | 5.44 (1.45 – 20.34) | 0.012 |
| >15 kPa | 48.52 (16.72 – 140.80) | <0.001 | 21.08 (6.49 – 68.48) | <0.001 |
| **Histology** |  |  |  |  |
| F0-1-2 | 1 (Reference) |  | 1 (Reference) |  |
| F3 | 7.68 (2.42 – 24.28) | 0.001 | 6.30 (1.69 – 23.44) | 0.006 |
| F4 | 80.50 (28.82 – 224.81) | <0.001 | 41.24 (11.70 – 145.44) | <0.001 |
| **FIB-4** |  |  |  |  |
| <1.3^†^ | 1 (Reference) |  | 1 (Reference) |  |
| 1.3^†^-2.67 | 8.51 (1.77 – 40.95) | 0.008 | 5.63 (1.14 – 27.75) | 0.034 |
| >2.67 | 85.76 (20.23 – 363.60) | <0.001 | 32.38 (6.84 – 153.31) | <0.001 |

Hazard ratios from univariable and multivariable Cox regression for prediction of secondary composite outcomes according to 3 groups of low, intermediate and high risk, with p values for between-group. Covariates of multivariable Cox regression are diabetes, age, gender and BMI. †2 for patients older than 65 years
