# Supplementary figures and images for: Enhanced Liver Fibrosis Test, FIB‐4 and FibroScan: Real‐World Prognostic Accuracy for MASLD in a Biopsy‐Controlled Cohort
Source: Liver Int. 2026 Jul 2;46(8):e70774. doi: 10.1111/liv.70774 (PMC13329087; doi:10.1111/liv.70774)

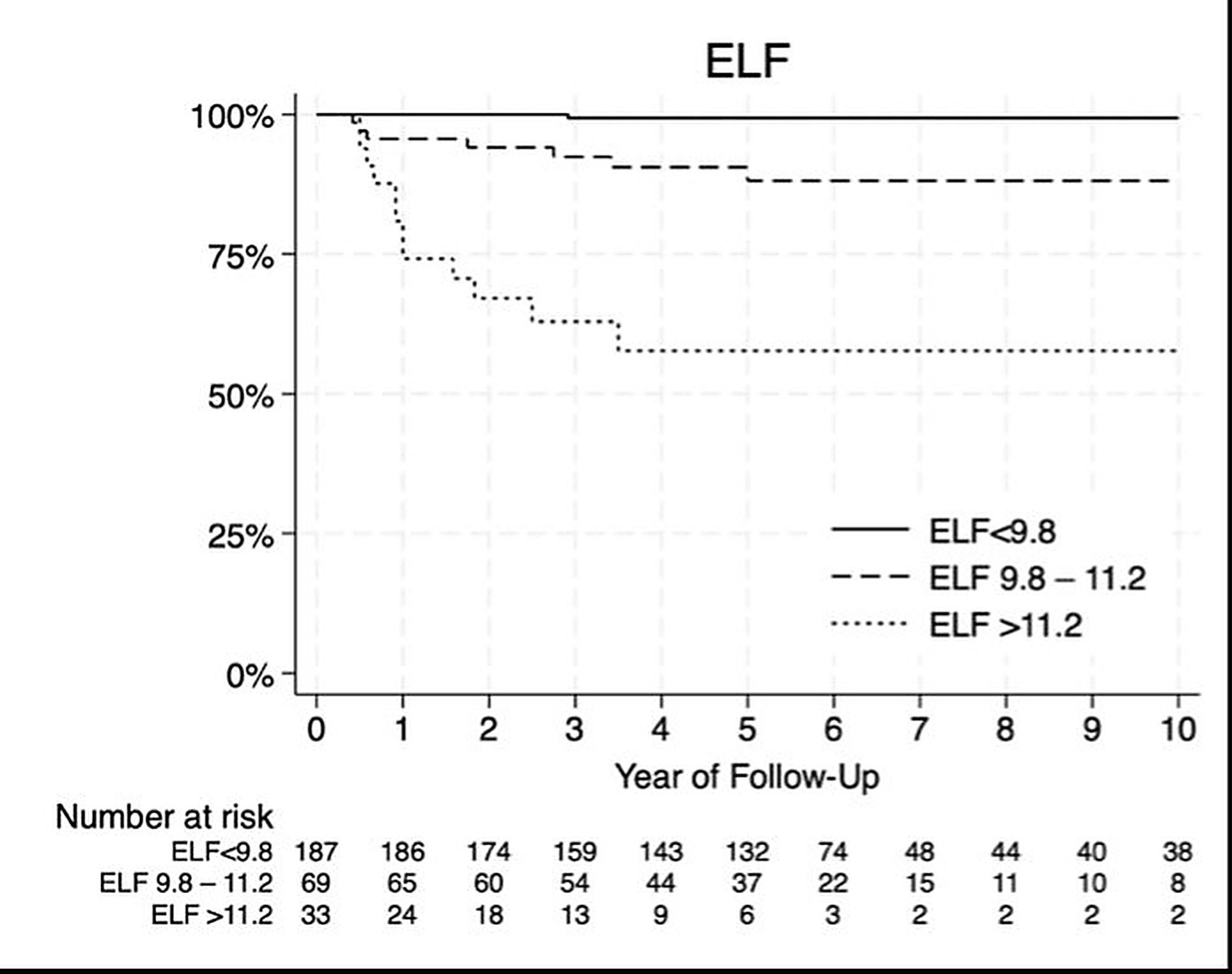

Supplement: Supplementary file 2 — Figure S1: Kaplan–Meier curves for time to AD according to histology, FIB‐4, LSM and ELF pre‐defined cut‐offs. Kaplan–Meier curves for time to the development of ascites (AD). Patients were stratified into low, intermediate, and high‐risk groups based on pre‐defined cutoffs for ELF, FIB‐4, histology, and LSM. Risk group definitions: High risk: ELF > 11.2, FIB‐4 > 2.67, Histology F4, LSM > 15 kPa; Intermediate risk: ELF 9.8–11.2, FIB‐4 1.3–2.67 (or 2.0–2.67 if age > 65 years), Histology F3, LSM 10–15 kPa; Low risk: ELF < 9.8, FIB‐4 < 1.3 (or < 2.0 if age > 65 years), Histology F0‐2, LSM < 10 kPa. Time is expressed in years. [file LIV-46-0-s003.zip › liv70774-sup-0003-FigureS1@SupplemFig_1_panel_B_elf_AD.png]

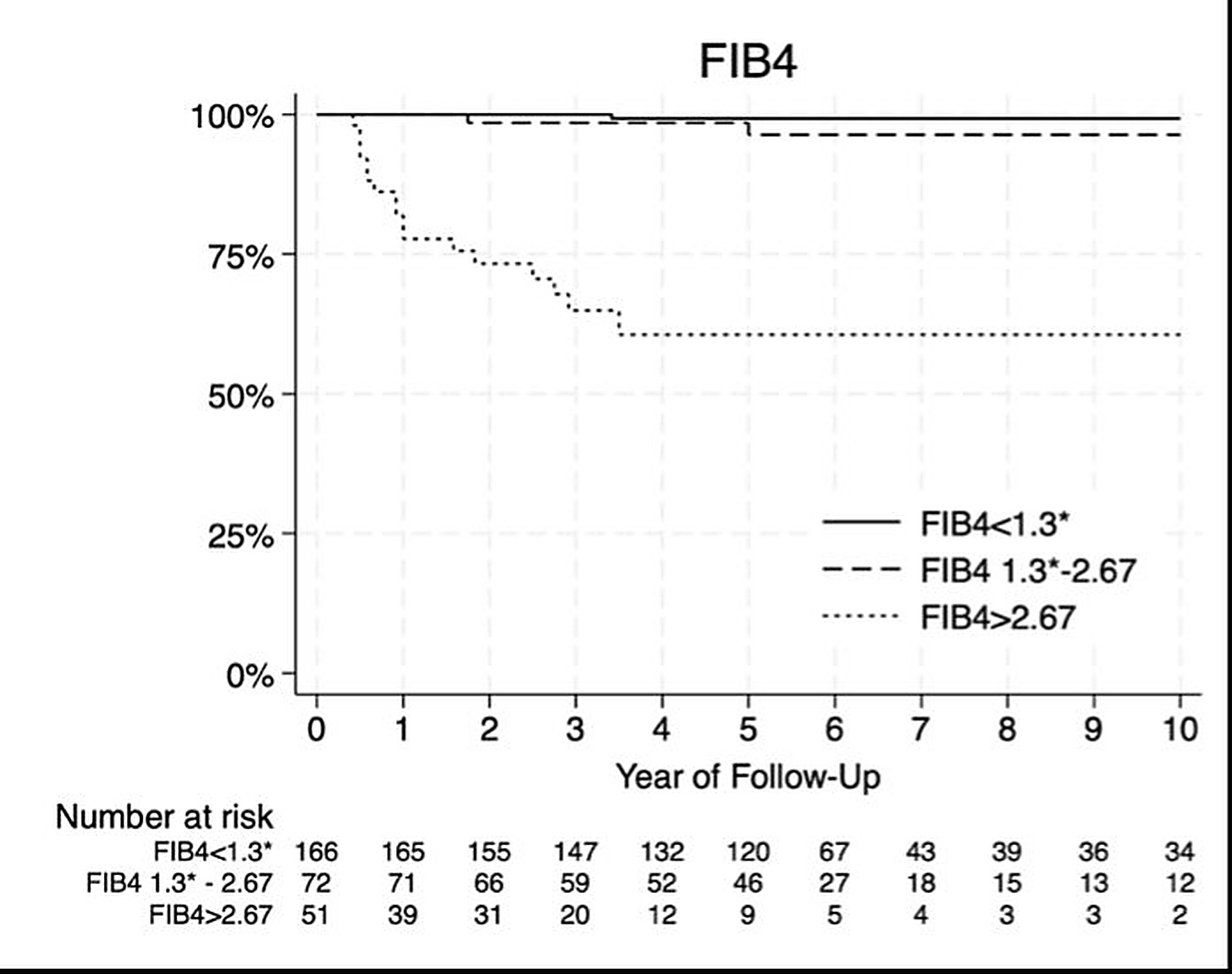

Supplement: Supplementary file 2 — Figure S1: Kaplan–Meier curves for time to AD according to histology, FIB‐4, LSM and ELF pre‐defined cut‐offs. Kaplan–Meier curves for time to the development of ascites (AD). Patients were stratified into low, intermediate, and high‐risk groups based on pre‐defined cutoffs for ELF, FIB‐4, histology, and LSM. Risk group definitions: High risk: ELF > 11.2, FIB‐4 > 2.67, Histology F4, LSM > 15 kPa; Intermediate risk: ELF 9.8–11.2, FIB‐4 1.3–2.67 (or 2.0–2.67 if age > 65 years), Histology F3, LSM 10–15 kPa; Low risk: ELF < 9.8, FIB‐4 < 1.3 (or < 2.0 if age > 65 years), Histology F0‐2, LSM < 10 kPa. Time is expressed in years. [file LIV-46-0-s003.zip › liv70774-sup-0004-FigureS1-S4@SupplemFig_1_panel_C_FIB4_AD.png]

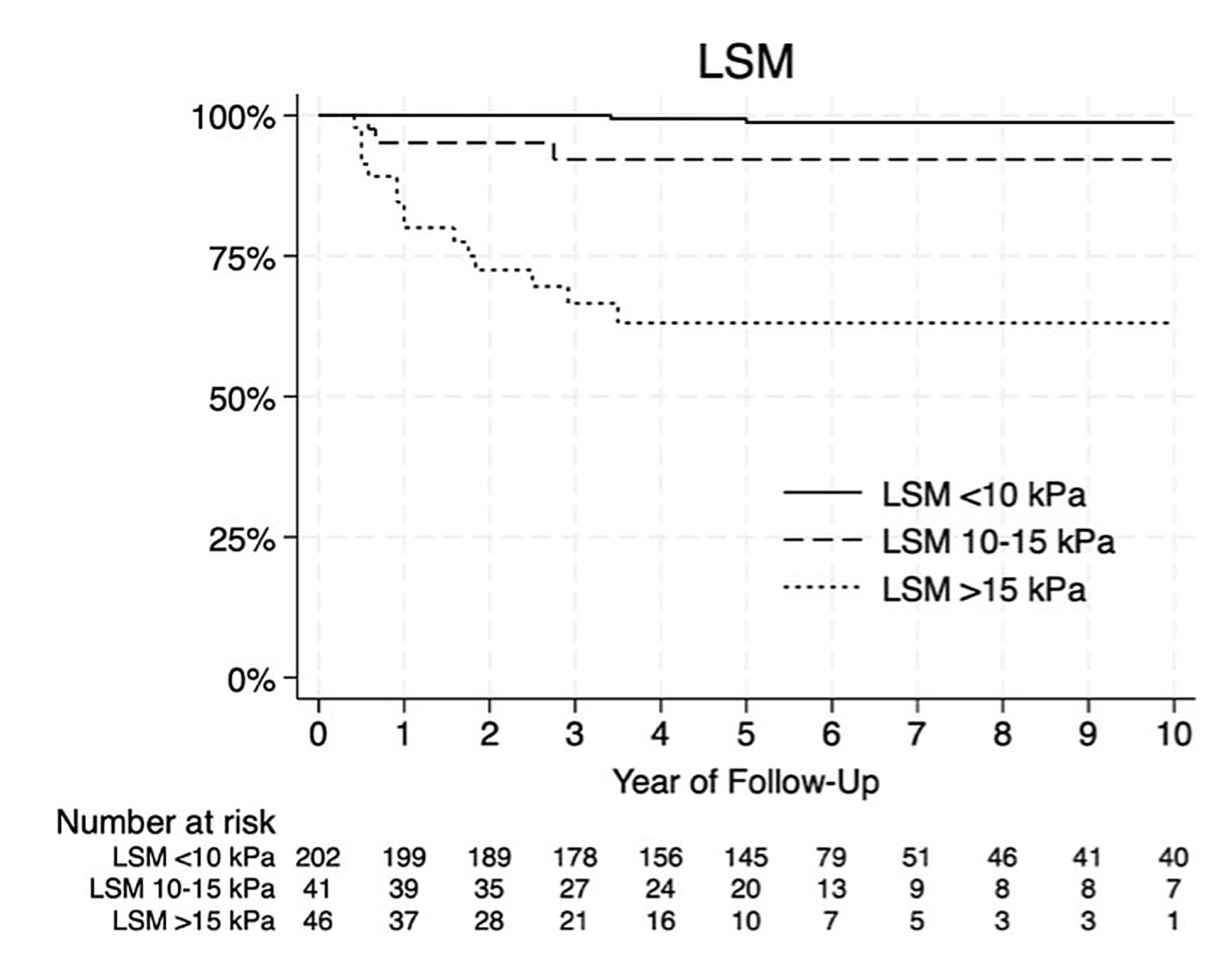

Supplement: Supplementary file 2 — Figure S1: Kaplan–Meier curves for time to AD according to histology, FIB‐4, LSM and ELF pre‐defined cut‐offs. Kaplan–Meier curves for time to the development of ascites (AD). Patients were stratified into low, intermediate, and high‐risk groups based on pre‐defined cutoffs for ELF, FIB‐4, histology, and LSM. Risk group definitions: High risk: ELF > 11.2, FIB‐4 > 2.67, Histology F4, LSM > 15 kPa; Intermediate risk: ELF 9.8–11.2, FIB‐4 1.3–2.67 (or 2.0–2.67 if age > 65 years), Histology F3, LSM 10–15 kPa; Low risk: ELF < 9.8, FIB‐4 < 1.3 (or < 2.0 if age > 65 years), Histology F0‐2, LSM < 10 kPa. Time is expressed in years. [file LIV-46-0-s003.zip › liv70774-sup-0005-FigureS1@SupplemFig_1_panel_D_LSM_AD.png]

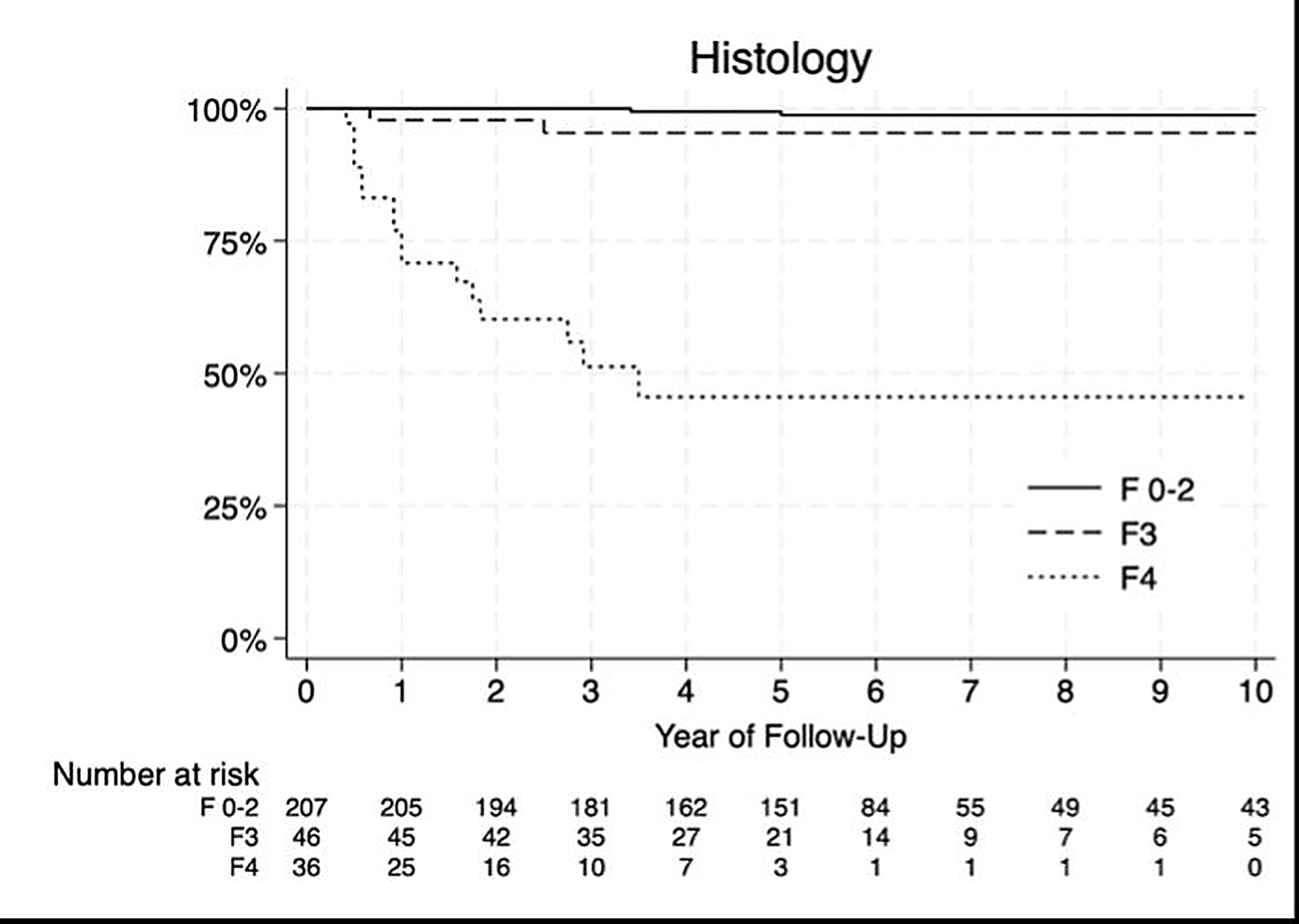

Supplement: Supplementary file 2 — Figure S1: Kaplan–Meier curves for time to AD according to histology, FIB‐4, LSM and ELF pre‐defined cut‐offs. Kaplan–Meier curves for time to the development of ascites (AD). Patients were stratified into low, intermediate, and high‐risk groups based on pre‐defined cutoffs for ELF, FIB‐4, histology, and LSM. Risk group definitions: High risk: ELF > 11.2, FIB‐4 > 2.67, Histology F4, LSM > 15 kPa; Intermediate risk: ELF 9.8–11.2, FIB‐4 1.3–2.67 (or 2.0–2.67 if age > 65 years), Histology F3, LSM 10–15 kPa; Low risk: ELF < 9.8, FIB‐4 < 1.3 (or < 2.0 if age > 65 years), Histology F0‐2, LSM < 10 kPa. Time is expressed in years. [file LIV-46-0-s003.zip › liv70774-sup-0002-FigureS1@SupplemFig_1_panel_A_Hist_AD.png]

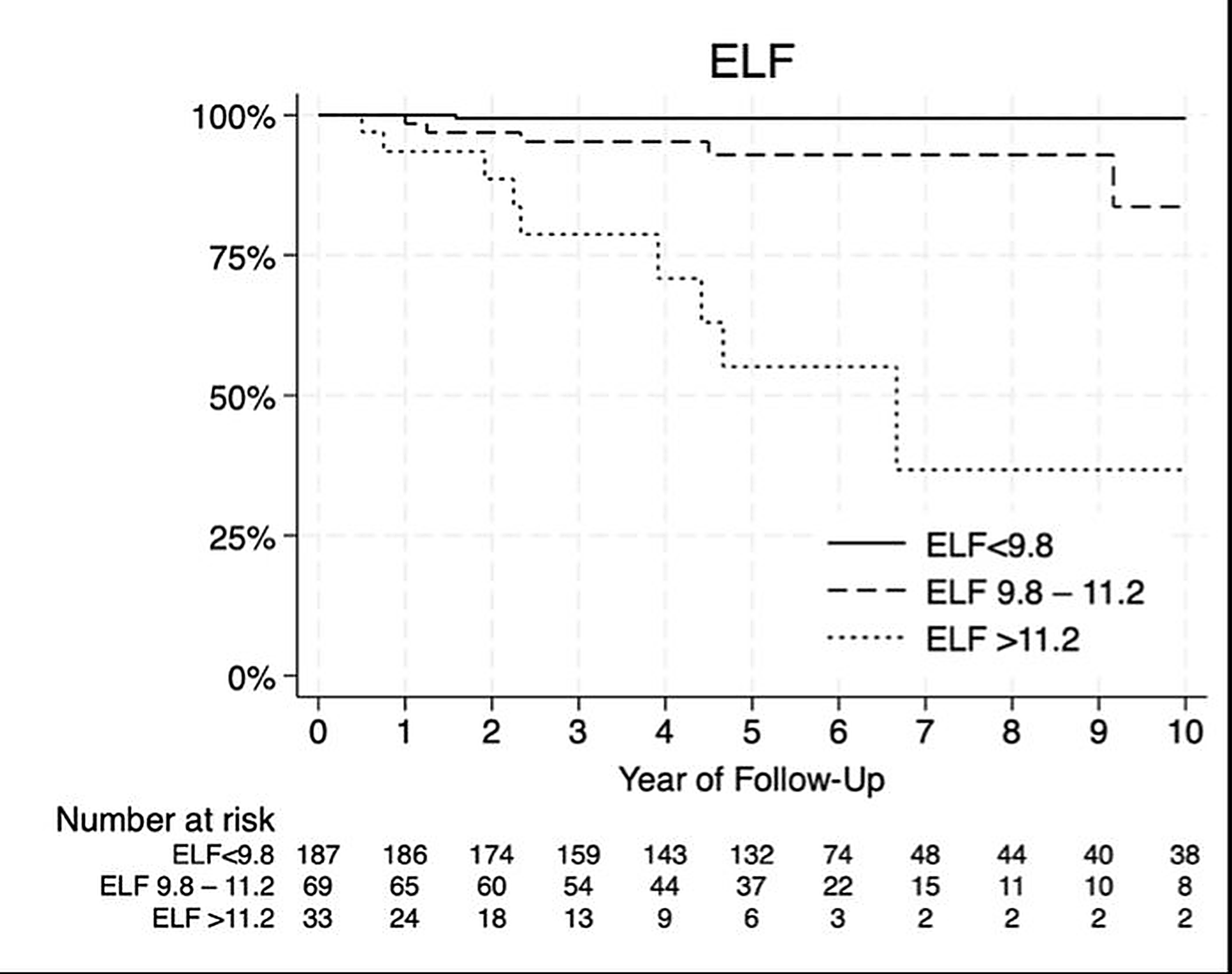

Supplement: Supplementary file 3 — Figure S2: Kaplan–Meier Curves for Time to ACLF According to Histology, FIB‐4, LSM and ELF Pre‐defined Cutoffs. Kaplan–Meier curves for time to the development of acute‐on‐chronic liver failure (ACLF). Patients were stratified into low, intermediate, and high‐risk groups based on pre‐defined cutoffs for ELF, FIB‐4, histology, and LSM. Risk Group Definitions: High Risk: ELF > 11.2, FIB‐4 > 2.67, Histology F4, LSM > 15 kPa; Intermediate Risk: ELF 9.8–11.2, FIB‐4 1.3–2.67 (or 2.0–2.67 if age > 65 years), Histology F3, LSM 10–15 kPa; Low Risk: ELF < 9.8, FIB‐4 < 1.3 (or < 2.0 if age > 65 years), Histology F0‐2, LSM < 10 kPa. Time is expressed in years. [file LIV-46-0-s001.zip › liv70774-sup-0007-FigureS2-S200@SupplemFig_2_panel_B_ELF_ACLF_200dpi.png]

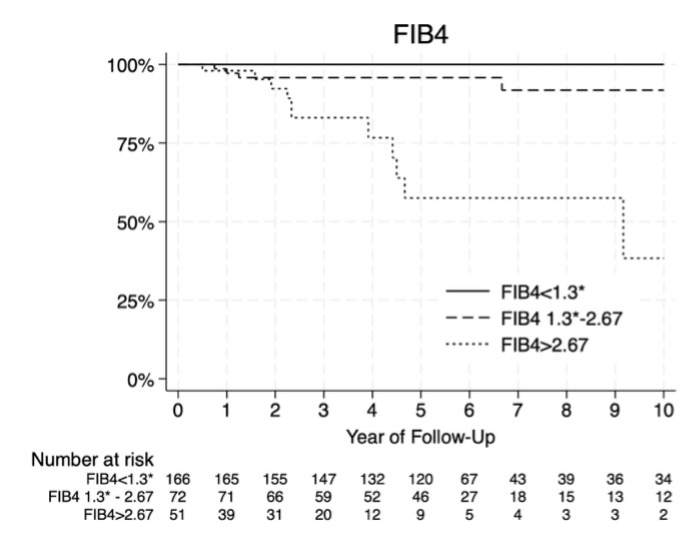

Supplement: Supplementary file 3 — Figure S2: Kaplan–Meier Curves for Time to ACLF According to Histology, FIB‐4, LSM and ELF Pre‐defined Cutoffs. Kaplan–Meier curves for time to the development of acute‐on‐chronic liver failure (ACLF). Patients were stratified into low, intermediate, and high‐risk groups based on pre‐defined cutoffs for ELF, FIB‐4, histology, and LSM. Risk Group Definitions: High Risk: ELF > 11.2, FIB‐4 > 2.67, Histology F4, LSM > 15 kPa; Intermediate Risk: ELF 9.8–11.2, FIB‐4 1.3–2.67 (or 2.0–2.67 if age > 65 years), Histology F3, LSM 10–15 kPa; Low Risk: ELF < 9.8, FIB‐4 < 1.3 (or < 2.0 if age > 65 years), Histology F0‐2, LSM < 10 kPa. Time is expressed in years. [file LIV-46-0-s001.zip › liv70774-sup-0008-FigureS2-S4@SupplemFig_2_panel_C_FIB4_ACLF.jpg]

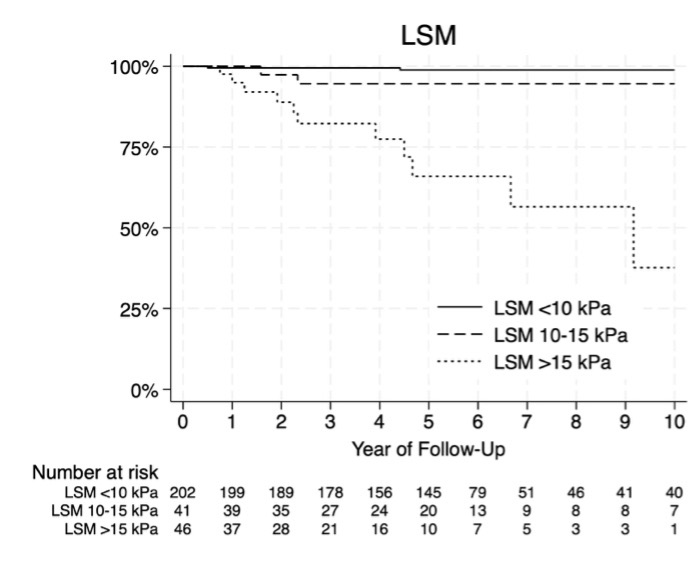

Supplement: Supplementary file 3 — Figure S2: Kaplan–Meier Curves for Time to ACLF According to Histology, FIB‐4, LSM and ELF Pre‐defined Cutoffs. Kaplan–Meier curves for time to the development of acute‐on‐chronic liver failure (ACLF). Patients were stratified into low, intermediate, and high‐risk groups based on pre‐defined cutoffs for ELF, FIB‐4, histology, and LSM. Risk Group Definitions: High Risk: ELF > 11.2, FIB‐4 > 2.67, Histology F4, LSM > 15 kPa; Intermediate Risk: ELF 9.8–11.2, FIB‐4 1.3–2.67 (or 2.0–2.67 if age > 65 years), Histology F3, LSM 10–15 kPa; Low Risk: ELF < 9.8, FIB‐4 < 1.3 (or < 2.0 if age > 65 years), Histology F0‐2, LSM < 10 kPa. Time is expressed in years. [file LIV-46-0-s001.zip › liv70774-sup-0009-FigureS2@SupplemFig_2_panel_D_LSM_ACLF.jpg]

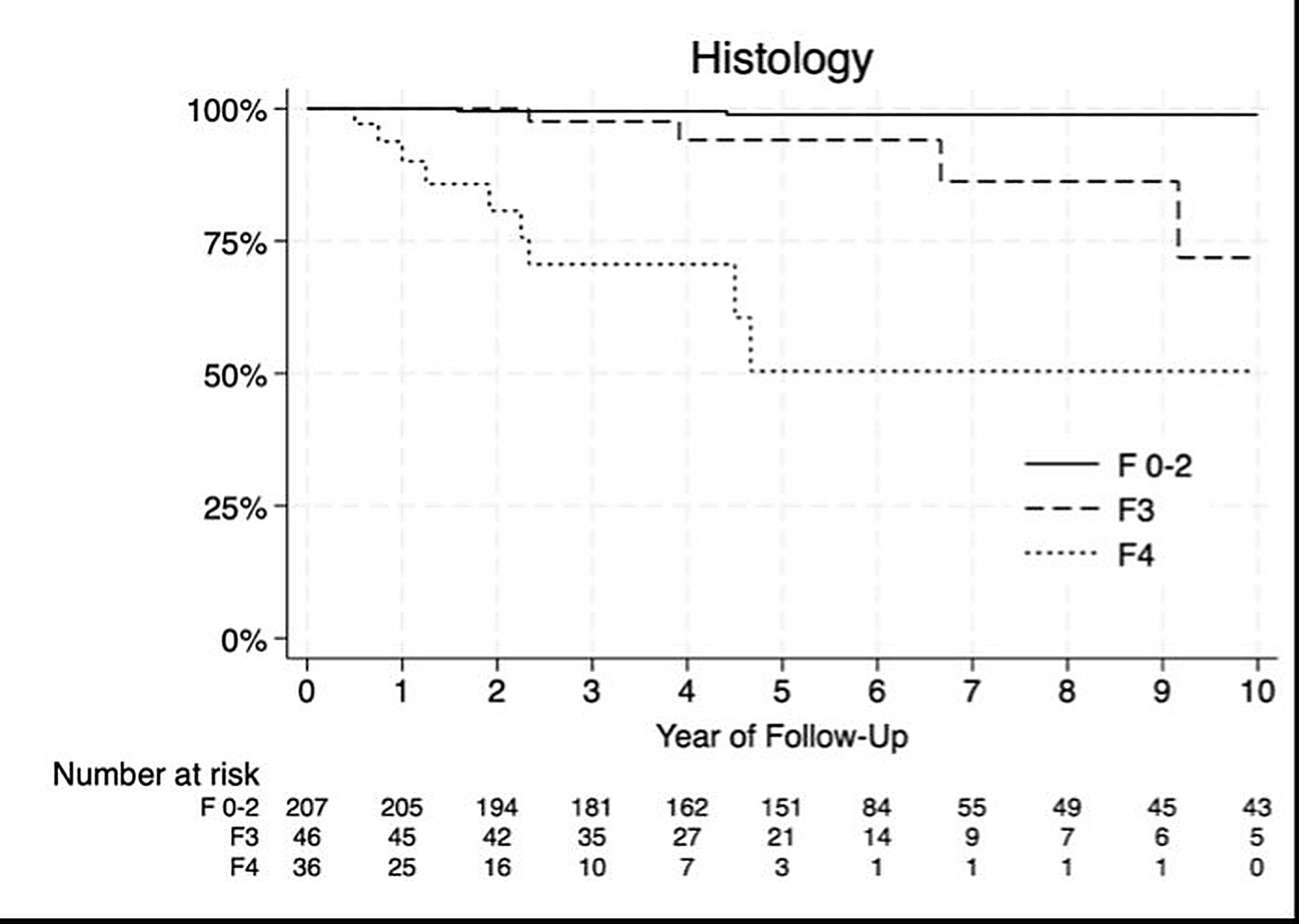

Supplement: Supplementary file 3 — Figure S2: Kaplan–Meier Curves for Time to ACLF According to Histology, FIB‐4, LSM and ELF Pre‐defined Cutoffs. Kaplan–Meier curves for time to the development of acute‐on‐chronic liver failure (ACLF). Patients were stratified into low, intermediate, and high‐risk groups based on pre‐defined cutoffs for ELF, FIB‐4, histology, and LSM. Risk Group Definitions: High Risk: ELF > 11.2, FIB‐4 > 2.67, Histology F4, LSM > 15 kPa; Intermediate Risk: ELF 9.8–11.2, FIB‐4 1.3–2.67 (or 2.0–2.67 if age > 65 years), Histology F3, LSM 10–15 kPa; Low Risk: ELF < 9.8, FIB‐4 < 1.3 (or < 2.0 if age > 65 years), Histology F0‐2, LSM < 10 kPa. Time is expressed in years. [file LIV-46-0-s001.zip › liv70774-sup-0006-FigureS2-S200@SupplemFig_2_panel_A_Hist_ACLF_200dpi.png]
